# Impact of MRSA Transmission and Infection in a Neonatal Intensive Care Unit in China: A Bundle Intervention Study during 2014-2017

**DOI:** 10.1155/2019/5490413

**Published:** 2019-07-10

**Authors:** Huiping Huang, Jing Ran, Jianzhou Yang, Peng Li, Guihua Zhuang

**Affiliations:** ^1^School of Public Health, Health Science Center, Xi'an Jiaotong University, 76 West Yanta Road, Xi'an 710061, China; ^2^Department of Infection Control, The First Affiliated Hospital of Xiamen University, 55 Zhenhai Road, Xiamen, 361003, China; ^3^Department of Gynecology and Obstetrics, The First Affiliated Hospital of Xiamen University, 55 Zhenhai Road, Xiamen, 361003, China

## Abstract

*Objective. *To evaluate the efficacy of bundle intervention on healthcare-associated (HA) methicillin-resistant* Staphylococcus Aureus *(MRSA) infection in the neonatal intensive care unit (NICU).* Methods. *In this study, 11,277 infants having undergone treatment at the NICU in Xiamen, China, from January 2014 to February 2017 were recruited. We retrospectively reviewed patients' demographic and clinical information. Patients from 2014 to 2015 were treated as the control group and those from 2016 to 2017 were classified as the experimental group. Bundle intervention measures were performed, including screening for MRSA, isolation precautions, training of hand hygiene, cleaning protocols, and decontamination of isolation ward. The HA-MRSA data and compliance of infection control measures between both groups were analyzed.* Results*. Through bundle interventions, the compliance with the isolation of MRSA raised from 55.88% to 92.86% and hand hygiene compliance increased from 90.07% to 93.23% (*P* < 0.05). The HA infection decreased from 1.87% to 1.71% (*P* > 0.05) and HA detection rate of MRSA declined from 2.63‰ to 1.00‰, respectively (*P* < 0.05).* Conclusion. *Multifaceted interventions can effectively prevent MRSA infection and transmission; this includes active surveillance, isolation precautions, increased hand hygiene compliance, environmental cleaning, and decontamination.

## 1. Introduction

Methicillin-resistant* Staphylococcus aureus* (MRSA) is one type of multidrug resistant organisms (MDROs) which can remain in the environment for a long term [1]. With the broad-spectrum antimicrobial widely used in clinical settings, incidences of MRSA infection are growing worldwide [2]. MRSA refers to a major healthcare-associated infection (HAI) organism associated with an increasing morbidity and mortality rate. As neonatal immunologic function is immature, MRSA infections occur more frequently in neonatal intensive care units (NICUs). MRSA is reported to be associated with the infection of skin and soft tissue (SSTI) as well as respiratory tracts. In the meantime, HA-MRSA is the main cause of pneumonia, osteomyelitis, and bacteremia [3, 4]. Most neonatal bloodstream infections (BSI) are MRSA-related sepsis which are associated with a high mortality rate [5–7]. HA-MRSA infections are more aggressive and difficult to diagnose and treat which leads to higher mortality rates, longer hospital stays, and increased financial burdens [8, 9].

The prevalence of MRSA is increasing in neonates [10, 11]. MRSA can be spread from environment to patients, and it can also be transmitted by colonized or infected patients [1, 12]. Before a proper cleaning, medical devices (stethoscopes, otoscopes, and thermometers) and various objects in hospital environments were positive for* Staphylococcus* spp., which has been associated with transmission of HAI [1]. MRSA is subject to a higher risk of transmission than MSSA [13, 14]. Control of HA-MRSA remains challenging in NICUs: the recent research identified contaminated or dirty wound operations and MRSA colonization during hospitalization as risk factors for SSI in neonates [15]. Studies demonstrated MRSA had become endemic in numerous NICUs and caused invasive disease and, in some cases, even death [16–18]. For instance, the majority of outbreaks (in six out of 10) in North London were infected with MRSA [19]. Stricter adherence to disinfection practices by healthcare professionals would help to alleviate outbreak and transmission.

The published research demonstrated that the incidence of MRSA in hospital settings had decreased steadily since 2005 in the US, and this decline may be due to increased attention to infection prevention [20]. Several recent studies also have shown that HA-MRSA decreased significantly after the implementation of the intervention methods [21, 22]. The Society for Healthcare Epidemiology of America (SHEA) has developed guidelines for the prevention of transmission of MRSA within healthcare settings; chief among the recommendations is to improve hand hygiene. Infection control measures were performed to prevent transmission and reduce the risk of acquiring HA-MRSA (e.g., isolation precautions, active surveillance, and hand hygiene compliance). However, whether these measures are effective or sufficient remains controversial [23–25]. Initiative through universal screening and isolation to prevent MRSA infections was considered cost effective [26]; however, universal surveillance results in an overall increase in costs [27]. A patient-centered care bundle intervention is effective but not cost efficient. MRSA are often transmitted through contact with healthcare personnel. The success of an infection control program requires bundle intervention, effective leadership, and a positive work environment. To achieve success, it also relies heavily on the cooperation and participation of healthcare personnel in both behavioral and practice changes [28].

The research studies of the bundle interventions for MRSA are limited, especially in developing countries [17]. The preventability of HA-MRSA in NICU's has rarely been evaluated via in-depth study. Thus, the intervention study was carried out here to explore MRSA transmission and infection trend in NICUs. Patients recruited in this study were from the NICU of a university-affiliated hospital with more than 2000 beds in Xiamen, China. We analyzed the compliance of multifaceted interventions, the clinical characteristics, and the MRSA infection trend. This study aimed to investigate the association of bundle interventions with the MRSA transmission and to provide effective infection control measures to prevent MRSA transmission in NICUs.

## 2. Materials and Methods

### 2.1. Setting and Design

The First Affiliated Hospital of Xiamen University is a Joint Commission International (JCI) accredited academic medical center hospital which acquired certification in 2015 and HIMSS EMRAM level 7 in 2017. It houses an 80-bed, Level III NICU. The NICU has 3 private rooms and 4 open bays. 11,277 neonates were admitted to the NICU between January 1, 2014, and December 31, 2017. The relationship between MRSA infection or colonization and these patients was analyzed based on those neonates. During this period, 5,305 neonates were retrospectively reviewed. A bundle intervention study was conducted covering active surveillance, isolation precautions, and hand hygiene promotion, and 5,972 neonates were hospitalized from January 1, 2016, to December 31, 2017.

The collected data, including the medical records of the cases, were extracted from the HAI real-time monitoring system connected with the electronic medical record and inspection system. Medical records were reviewed: this included the demographics, clinical care, microbiologic data, antibiotic resistance, isolation order, and outcomes of the patients with infection or colonization MRSA.

### 2.2. Surveillance and Cultures

Following the internal University Medicine Goettingen (UMG) guidelines, neonates having high risk factors of MRSA carriers were contacted in isolation, placed in a private room, and then screened for MRSA on admission. The neonate's mother then acts as a potential carrier of the infectious disease and has transferred the infection between hospitals and becomes the high-risk factor for the development of MRSA. Samples of nasal secretions were obtained with a swab for MRSA screened within 24 hours after their admission to the NICU. Bacterial isolation and antimicrobial susceptibility testing were performed in accordance with the methodology of the Clinical and Laboratory Standards Institute [29]. Using the disk diffusion method with S aureus ATCC 25923 as the quality control strain, susceptibility testing was routinely performed. The isolated* Staphylococcus aureus* was cultured with oxacillin at 2*μ*g/ml. If* Staphylococcus aureus* could grow in oxacillin at a concentration greater than 2*μ*g/ml, it was determined to be MRSA.

WHO's (World Health Organization's) "Five Moments for Hand Hygiene" was adopted to assess healthcare workers hand hygiene compliance. Two infection control nurses evaluated hand hygiene compliance though direct observation. The observers remained unchanged during the study. In addition, the contact isolation compliance with MRSA infection or colonization neonates were monitored by infection control nurses. HAIs were verified in accordance with the National Healthcare Safety Network's (NHSN's) surveillance definitions by the Centers for Disease Control and Prevention [30].

### 2.3. Bundle Interventions

Newborns whose mothers were diagnosed with infection and those transferred from other hospitals were the high-risk factors for the development of MRSA carriers. Accordingly, they would be screened for MRSA. They were placed on contact precautions in a private room following standard precautions until pathogen culture results were reported.

To identify the colonized or infected neonates with MRSA, the doctors in charge should prescribe contact isolation to start the bundle interventions. There are many ways to isolate patients via nurses (e.g., pathogen isolation, alcohol disinfection of the bed, hanging blue contact isolation mark on the door and bed of isolation ward, “MRSA” sign on the patient's electronic medical record, and yellow button on the wristband). Only after all the measures are completed would it be considered a successful intervention. Fractions were used to calculate the intervention success rate. The denominator is the number of patients who should be isolated, and the numerator is the number of patients receiving successful intervention. The medical supplies of each neonate with MRSA were used solely for that patient (e.g., stethoscope). The bed unit was wiped by nurses with disinfectant three times a day. HCWs (healthcare workers) were trained on proper prevention and how to best control MRSA transmission in a hospital setting, and hand hygiene activities were promoted to improve the hand hygiene compliance of HCWs.

The compliance of contact isolation was supervised by infection control nurses and the implementation was supervised by the infection control professional weekly. The bundle interventions also constructed a supervision mechanism for the medical staff. An official automatic (OA) network would record and report monthly incidents of misconduct where doctors did not prescribe contact isolation advice or nurses did not implement contact isolation. These misconducts were calculated into performance appraisal.

Major monitoring indicators covered the following:

- MRSA detection rate of* Staphylococcus aureus*
- The incidence of events of colonization or infection with MRSA neonates
- Rates of healthcare-associated MRSA infections per 1, 000 inpatients in NICU
- Rates of community-acquired MRSA infections per 1, 000 inpatients in NICU
- The hand hygiene compliance of healthcare workers

### 2.4. Statistical Analysis

In this study, the statistical analyses were performed using SPSS 13.0 (SPSS Inc., Chicago, IL, USA). The continuous variables were presented as mean ± standard deviation (mean ± SD), and the categorical variables were expressed as number and percentage (%). Continuous variables were analyzed using Student's* t*-test and categorical variables were analyzed using the *χ*^2^ test. Pearson's correlation analysis was performed to investigate the relationship between the rate of HA-MRSA and the rate of contact isolation, HAI, and hand hygiene. All* P *values were 2 tailed, and* P* < 0.05 was considered as significantly different.

## 3. Results

### 3.1. Clinical Characteristics of MRSA Colonized or Infected Neonates

During the study period, 11,277 patients were admitted to the NICU. Among all patients, males made up 54.90%, females made up 45.10%, and 3,383 (30.00%) were either transferred from another hospital or admitted from home. The median length of stay in the NICU was 9.65 ± 10.63 days (range 1-97). 261 neonates (2.31%) had a culture grow* Staphylococcus aureus* and the median age was 15.73 ± 8.05 days. The detection rate of MRSA was 29.12% (76/261) among total isolates of* Staphylococcus aureus*. The median length of stay in the NICU of the neonates with methicillin-susceptible* S. aureus *(MSSA) reached 8.68 ± 8.08 days. MRSA was 14.26 ± 17.91 days. The median length of MRSA was significantly longer than MSSA (t = 2.83, P = 0.005). MRSA infections covered 49 lower respiratory infections (64.47%), 16 skin and soft tissue infections (21.05%), 7 gastrointestinal infections (9.21%), 3 bacteremia, and 1 ventilator-associated pneumonia (VAP). Two of the 76 infected neonates (2.63%) died but only one was due to an MRSA infection. From the periods of 2014-2015 and of 2016-2017, incidents of MRSA in NICUs increased from 6.41‰ (34/5305) to 7.03‰ (42/5972); there was no significant difference (*χ*^2^ = 0.16,* P* = 0.686). The methicillin resistance rate of* Staphylococcus aureus* rose from 25.37% (34/134) to 33.07% (42/127) after implementing bundle interventions; it was not significantly lower (*χ*^2^ = 2.68,* P* = 0.102), as shown in Table 1.

### 3.2. The Compliance of MRSA Contact Isolation

After implementing bundle interventions the compliance of MRSA contact isolation grew from 55.88% to 92.86% which was significantly higher than before (*χ*^2^ = 14.21, P=0.001), as shown in Table 2.

### 3.3. The Compliance of Hand Hygiene in NICUs

The compliance of hand hygiene before intervention from 2014 to 2015 reached 90.07% (363/403) and, after intervention from 2016 to 2017, the compliance of hand hygiene rose to 93.23% (909/975). It was significantly higher (*χ*^2^ = 4.00,* P* = 0.045), as shown in Table 3.

### 3.4. HAI and MRSA Infection of Neonates

The rate of HAI in NICUs dropped from 1.87% to 1.71% after intervention; there was no significant difference (*χ*^2^ = 0.41, P = 0.524). Median length of stay of the neonates with hospital-acquired MRSA after admission was 11.25 ± 6.49 days (range 4-21). The rate of hospital-acquired MRSA after intervention decreased from 2.63‰ to 1.00‰. It was significantly lower (*χ*^2^ = 4.24, P = 0.04). The rate of community-acquired (CA) MRSA increased from 3.77‰ to 6.03‰, therefore displaying no significant difference (*χ*^2^ = 2.90, P = 0.089), as shown in Table 4.

### 3.5. Isolated Strains of HAI

Among the 201 isolated strains, the main strains included 46* K. pneumoniae*, 35* P. aeruginosa*, and 28* A. baumannii*. The most frequently isolated specimens were sputum (45.27%), blood (22.88%), urine (8.46%), pus (3.98%), etc. The isolated specimen from pus declined from 5.05% to 2.94% after the intervention, and no difference existed in the sequence of main infection sites.

### 3.6. Correlation between the Rate of Hospital-Acquired MRSA and Contact Isolation Compliance, HAI, and Hand Hygiene Compliance

To find the relationship between the rate of hospital-acquired MRSA, contact isolation compliance, HAI, and hand hygiene compliance, a total of 48 months of data (quarter of a year) were studied. The rate of hospital-acquired MRSA was significantly correlated with contact isolation compliance (r = -0.888, P <0.01), and the rate of hospital-acquired MRSA was not significantly correlated with that of HAI (r = 0.172, P = 0.525) or hand hygiene compliance (r = -0.311, P = 0.241).

## 4. Discussion

Some studies have reported that there is a decreasing incidence in MRSA among hospitalized adults in the United States in recent years [31]. Dantes et al. estimated a 54% decline in invasive MRSA [20]. However, MRSA is a common etiological agent of a life-threatening infection in NICUs, with increasing in-hospital mortality rates and prolonging hospital length of stay [3]. In this study, length of stay showed statistically significant differences between MRSA colonized neonates and MSSA colonized neonates and the similar results are shown in the previous report by Geraci et al. [32].

Our study also showed that lower respiratory tract infections with MRSA made up 64.47% while skin and soft tissue infections made up 21.05%. Some studies have reported that MRSA was mostly isolated from blood and bronchoalveolar lavage [3, 33]. Li et al. reported pneumonia (69, 53.1%) was the most common infection of CA-MRSA in Chinese neonates [34]. Of 11,277 neonates, 76 (6.74‰) had a culture grow MRSA including 20 neonates with NICU-acquired MRSA. This was lower than that of other studies which was, respectively, reported as 2% and 5.8% [12, 35].

The annual incidence density of acquisition of MRSA ranged from 5.66 cases to 7.66 cases per 1000 admissions. This was lower than the 6.99% reported by Shirai et al. [3]. Geraci et al. reported that the acquisition of MRSA ranged from a maximum of 20.2 cases for 1000 patient-days to a minimum of 8.8 cases [32]. Infection surveillance is managed by the real-time infection monitor system in hospitals since 2009. The underreporting potential nosocomial infections and MRSA infection were not found in the ward during the study. MRSA infection was lower with the contributing factor being the characteristic difference between western populations and Chinese. The methicillin resistance rate of* Staphylococcus aureus* ranged from 23.53% to 34.48%. HA-MRSA were more likely to develop into severe invasive infections. These infections could even cause death. In this study, one death was attributed to HA- MRSA infection. Harik et al. reported that 68% of MRSA cases were hospital-associated (HA) MRSA [36].

There was a relative risk of 24.2 for colonized patients in NICU to develop an MRSA infection during hospitalization [35]. MRSA contamination from high-touch surfaces is worrisome in developing countries [37]. A bundle of interventions are required in order for NICUs to prevent MRSA transmission. Single-room isolation is strongly recommended for neonates with high risk factors of MRSA.

Compliance with hand hygiene, active surveillance, and contact isolation either singularly or in conjunction have been insufficient and controversial. One study showed the decrease in MRSA acquisition was primarily attributed to the barrier effects of gowns and gloves followed by improved hand hygiene and lower HCW-patient contact rates [38]. However, other research reported only before and after patient contact rose from 40% to 76% for hand hygiene compliance. HAI and MRSA rates remained high and stable and there was no correlation between compliance and MRSA [25, 39].

A program of universal surveillance, contact precautions, hand hygiene, and institutional culture change was associated with the decrease in healthcare-associated transmissions of and infections with MRSA in an extensive healthcare system [21]. In our study, while the rates of healthcare-associated MRSA infections in NICUs had not changed during the two years before the intervention, they declined under the implementation of the bundle from 2.45 infections per 1000 admissions to 1.17 per 1000 admissions. During the same period, healthcare-associated infections declined slightly from 1.87% to 1.71%. This was because the main infection sites of healthcare-associated infections (including respiratory tract, bacteremia, and urinary tract) and the main pathogens (including* Klebsiella pneumoniae, Acinetobacter baumannii,* and* Pseudomonas aeruginosa)* are Gram-negative conditional pathogens that adapt to the surface of a moist environment and are easy to cause respiratory infection in patients with low immunity. MRSA is a Gram-positive bacterium which makes it easy to colonize on the surface of the human body and spreads through hand contact which is the primary cause of SSTIs.

Accompanied by the implementation of bundle interventions, both hand hygiene compliance and contact isolation grew significantly. Hand hygiene compliance was improved from 90.07% to 93.23% and MRSA contact isolation increased from 55.88% to 92.86%. The major cause of noncompliance was the lack of single room to isolate patients. By analyzing the correlations between the rate of hospital-acquired MRSA, contact isolation compliance, HAI, and hand hygiene compliance, we found that the rate of hospital-acquired MRSA was significantly correlated with that of contact isolation compliance. There was no significant correlation between the rate of hospital-acquired MRSA, HAI, and hand hygiene compliance. This revealed that screening for MRSA in addition to isolation precautions in MRSA bundle interventions are important and effective measures.

## 5. Conclusions

MRSA bundle interventions are capable of reducing healthcare-associated MRSA infections in NICUs. All research findings show that the bundle interventions are highly effective tools in MRSA prevention and should be adhered to routinely in order to improve contact isolation compliance.

## Figures and Tables

**Table 1:** Comparison of incidence and resistance rate of MRSA.

Year	Admissions	Neonates with MRSA isolates	Incidence of MRSA ‰	Staphylococcus aureus	Resistance rate of MRSA %
2014	2826	16	5.66	68	23.53
2015	2479	18	7.26	66	27.27
2016	2872	22	7.66	69	31.88
2017	3100	20	6.45	58	34.48

**Table 2:** The compliance of MRSA contact isolation.

Year	MRSA Neonates	Neonates in a private room	Ordered contact isolation	Marked as isolation	Number in compliance	Rate of compliance%
2014	16	10	12	12	10	62.50
2015	18	9	11	13	9	50.00
2016	22	20	22	22	20	90.91
2017	20	19	20	19	19	95.00

**Table 3:** Compliance of hand hygiene in NICUs.

Year	Moments of hand hygiene	Hand hygiene practices	Compliance %
2014	189	167	88.35%
2015	214	196	91.59%
2016	346	318	91.91%
2017	629	591	93.96%

**Table 4:** HAI and MRSA infection in NICUs.

Year	Admissions	Cases of HAI	Rate of HAI%	MRSA			
				HA		CA	
				Cases	Rate‰	Cases	Rate‰
2014	2826	50	1.77	6	2.12	10	3.54
2015	2479	49	1.98	8	3.23	10	4.03
2016	2872	39	1.36	4	1.39	18	6.26
2017	3100	63	2.03	2	0.64	18	5.81

## Data Availability

The data used to support the findings of this study are included within the article.
